# Chronotypes, Sleep and Mental Distress Among Chinese College Students: A Cross-Sectional Study

**DOI:** 10.3389/fpsyt.2022.883484

**Published:** 2022-05-26

**Authors:** Jiajia Wang, Shuai Liu, Junlong Guo, Rong Xiao, Jia Yu, Xian Luo, Yan Xu, Yuhan Zhao, Yingru Cui, Yue Gu, Lidan Cai, Bin Zhang

**Affiliations:** ^1^Department of Psychiatry, Nanfang Hospital, Southern Medical University, Guangzhou, China; ^2^Department of Psychology, School of Public Health, Southern Medical University, Guangzhou, China; ^3^Department of Psychosomatic Diseases, The Third People's Hospital of Panzhihua, Panzhihua, China

**Keywords:** chronotype, sleep compensation, insomnia, depression, anxiety

## Abstract

**Objective:**

This study aimed to investigate the chronotypes and their relationship with sleep disturbances and mental distress among college students.

**Methods:**

Students from a university in Guangzhou, China, were recruited through a cross-sectional online survey. Data were collected by self-reported questionnaires including socio-demographics, lifestyles and health conditions, sleep patterns on weekdays and weekends, as well as the reduced Morningness-Eveningness Questionnaire (rMEQ), the Insomnia Severity Index, the Epworth Sleepiness Scale, the Beck Depression Inventory-13, and the Zung Self-Rating Anxiety Scale. Multivariate analyses were performed to examine the associations of chronotypes with sleep compensation, sleep disturbances, and mental distress.

**Results:**

A total of 1,607 questionnaires were received, among which 1,569 (97.6%) were valid for further analysis. Among these participants [mean age 19.86 ± 1.16 (15–27) years], morning types (M-types), intermediate types (I-types), and evening types (E-types) accounted for 14.9, 71.5, and 13.6%, respectively. The regression analysis revealed that E-types were positively associated with long sleep compensation on weekends (adjusted OR 2.443, 95%CI 1.740-3.429) compared with I-types, while M-types were the opposite (adjusted OR 0.623, 95%CI 0.392–0.990). E-types were also positively correlated with insomnia symptoms (adjusted OR 2.000, 95%CI 1.428–2.801), depressive symptoms (adjusted OR 2.068, 95%CI 1.496–2.858), and anxiety symptoms (adjusted OR 2.188, 95%CI 1.387–3.451). However, no significant association was found between chronotypes and excessive daytime sleepiness.

**Conclusion:**

Our study found that E-types were associated with long sleep compensation on weekends and insomnia, depression, and anxiety symptoms. Our findings emphasized the importance of early recognition and intervention of E-types and their accompanied sleep problems and mental distress.

## Introduction

Chronotype refers to an individual's endogenous circadian rhythms and how it embeds into the 24-h day, which received increasing attention in the last decades (1, 2). There are three different categories of chronotypes: the morning type (M-type), the evening type (E-type), and the intermediate type (I-type) (3). Evidence demonstrated that chronotype widely affects physiology, cognition, and behavior (4). M-type individuals get up early and reach their peak of cognitive and physical performance in the morning compared with E-types who prefer later bed and wake times. Besides, M-type people find it difficult to stay awake at late-night hours, while E-type people plan their daily activities for the afternoon or evening and reach their peak performance later. Although the distinction of chronotypes is quite easy, most people are I-types rather than at the ends of this continuum (5). The individual differences in chronotypes seem to be influenced by age (6), gender (7), inherited (8), perinatal (9), and environmental factors to some extent (10).

Previous studies found that in addition to differences in sleep timing between different chronotypes, E-types had a shorter time in bed during the weekdays and slept longer on weekends than M-types (11). E-types were also associated with a greater likelihood of sleep disturbances, including insomnia (12, 13). Although previous research suggested that E-types were associated with poor sleep and daytime sleepiness (14), few studies investigated the chronotype with sleep disturbance and the sleep pattern of different chronotypes on weekdays and weekends among Chinese college students. A previous study in China indicated that E-type college students were associated with more sleep difficulties (15). Several studies showed that E-types were associated with increased risk for a constellation of negative health outcomes, including mental and physical health problems (16, 17). For instance, a meta-analysis (18) found a positive association between E-types and depressive symptoms in longitudinal and cross-sectional studies. However, the relationship between chronotypes and anxiety symptoms was inconsistent. Some research found that E-types are related to elevation in anxiety symptoms (19, 20), whereas another did not find a relationship between E-types and anxiety (13). Thus, the association between chronotype and mental distress remains unclear and warrants further investigation.

College students who form a subset of the world's population appear to be more vulnerable to sleep disturbance and mental distress due to the exhaustive routine of studies and extracurricular activities. Given that E-types are associated with many adverse effects on physical and mental health, we investigated the association of chronotypes with sleep disturbances and mental distress among Chinese college students. Therefore, we aimed to describe the contemporary chronotype distribution in a large sample of college students in the present work. We further aimed to describe sleep patterns, sleep disturbance, and mental distress with chronotypes in order to provide evidence for further prevention and control of sleep disturbance and mental distress in college students.

## Methods

### Participants

This survey was part of a school-based cross-sectional study conducted at a university in Guangzhou from December 2019 to January 2020. Participants who met the following criteria were included in the study: (1) undergraduate students studying in the university; (2) WeChat users; (3) volunteers to participate in this survey. Using the method of convenient sampling, 1,607 college students in Guangzhou completed the questionnaires compiled by the Questionnaire Star platform relying on WeChat. This study has been approved by the Ethics Committee of Nanfang Hospital, Southern Medical University (QTEC-2019-101).

### Instruments

#### Socio-Demographics, Lifestyles, and Health Conditions

At the first of the questionnaire, the socio-demographic data were collected, including gender, grade, ethnicity, place of residence, whether they are the only child or not, their parents' education level (tertiary education or above), and family incomes (<5,000 yuan/month or ≥5,000 yuan/month). Lifestyles and health conditions included body-mass index (BMI, ≥25 kg/m^2^ or <25 kg/m^2^), habitual napping (≥3 days/week), habitual snoring (≥3 days/week), chronic medical conditions, smoking, alcohol drinking, boarding in school, time spent on TV or Internet (≥4 or <4 h/day), duration of mobile phone use before sleep (≥1 or <1 h/day), perceived learning pressure (high or low), learning interest (high or low).

#### Sleep Patterns and Weekend Sleep Compensation

Sleep patterns were collected, including habitual bedtimes and wake-up times on weekdays and weekends. Weekend sleep compensation was calculated as the difference between time in bed on weekends and weekdays, and long weekend sleep compensation was defined as compensation ≥2 h (21).

#### Reduced Morningness-Eveningness Questionnaire (rMEQ)

The reduced Morningness-Eveningness Questionnaire (rMEQ), developed by Adan and Almirall, is the most convenient and commonly used method to assess chronotype. It extracted five items from the original MEQ (items 1, 7, 10, 18, and 19) (5, 22) to assess the morningness-eveningness preference. The overall scores range from 4 to 25, with lower scores meaning a stronger preference for E-types. Participants whose scores were higher than 17 and lower than 12 were classified as morning and evening types, respectively, and participants scoring between 12 and 17 were classified as I-types. The rMEQ has been verified in the Chinese population and was proved to have good reliability and validity (Cronbach's alpha = 0.74) (23, 24).

#### Insomnia Severity Index (ISI)

The ISI comprises seven items that evaluate insomnia subtypes and daytime dysfunction due to sleep difficulties in the past 2 weeks. The index is rated on a 5-point Likert scale (0 = completely ineffective, 4 = very serious), and the total scores range from 0 to 28 (25, 26). A total score of ≥8 is considered to have insomnia symptoms. The internal reliability of the Chinese version of ISI was great (Cronbach's alpha = 0.8) (27).

#### Epworth Sleepiness Scale (ESS)

This scale assesses individuals' subjective daytime sleepiness and consists of seven items using a 4-point Likert scale. The sum score of the scale ranges from 0 (no chance of dozing) to 24 (highest chance of dozing). Excessive daytime sleepiness is defined as having a total score of 10 or higher (28). The Chinese version of ESS is reliable and well-validated (Cronbach's alpha = 0.81) (29).

#### Beck Depression Inventory-13 (BDI-13)

The BDI-13 is a 13-item scale evaluating the severity of depressive symptoms in the past week (30). A higher total score indicates a more severe depressive symptom. Each item is rated by a 4-point Likert scale (0 = none, 3 = severe), yielding a total score of 0 to 39. A total score of ≥5 was considered to experience depressive symptoms. The BDI-13 has been demonstrated good reliability and validity in previous studies of the Chinese population (Cronbach's alpha = 0.89) (31).

#### Zung Self-Rated Anxiety Scale (SAS)

The SAS is used to measure the anxiety symptoms in the past seven days (32). It consists of 20 items, each rated on a 4-point Likert scale (e.g., 1 = never or a little of the time, 4 = most of the time). The total score ranges from 25 to 100, multiplying the original score by 1.25. A total score of ≥50 was regarded as suffering from anxiety symptoms. The Chinese version of the questionnaire has been widely used and has satisfactory reliability and validity (Cronbach's alpha =0.78) (33).

### Statistical Analysis

The characteristics and distribution of chronotypes were described by descriptive statistics. Continuous variables were expressed as the mean ± standard deviation (SD), and non-normally distributed variables were expressed as median (interquartile range [IQR]), while the discrete variables were reported as percentages. In univariate analyses, differences between categorical variables were assessed using the chi-squared test. Statistical intergroup differences were analyzed using the *t*-test, one-way ANOVA, and Kruskal–Wallis test, which examined the association of socio-demographics, lifestyles, health conditions with sleep patterns, sleep disturbances, and mental distress. The Bonferroni *post-hoc* test for multiple comparisons was used for statistical analysis when necessary. Those factors significantly associated with sleep disturbances and mental distress would be further controlled in binary logistic regression models. In multivariate analyses, after adjusting for age and gender using the ENTER method, as well as other socio-demographics, lifestyles, and health conditions that had a significant difference in univariate analyses by using the forward likelihood method, binary logistic regression was performed to determine the strengths of the relationships between chronotypes and sleep compensation, sleep disturbances, and mental distress.

## Results

### General Characteristics of Participants

A total of 1607 questionnaires were received, among which 1,569 (97.6%) were valid for further analysis. Table 1 shows the socio-demographic and lifestyle characteristic of all valid participants. The participants consisted of 681 males (43.4%) and 888 females (56.6%), with an average age of 19.86 ± 1.16 years old (15–27 years old). Based on the score of rMEQ, 14.9% of the participants were found to be M-types, 71.5% were I-types, and 13.6% were E-types. In comparison of these three groups, differences were found significant in only child situation, rural area, family incomes, time spent on TV or Internet, duration of mobile phone use before sleep, alcohol drinking, chronic medical conditions, and interest in study (all *p* <0.05).

**Table 1:** Demographic and lifestyle characteristics of participants stratified by chronotypes.

	**Total (*n* = 1,569)**	**M-types (*n* = 233)**	**I-types (*n* = 1,122)**	**E-types (*n* = 214)**	***P***
**Socio-demographics**
Age, year, (Mean ± SD)	19.86 ± 1.16	19.80 ± 1.31	19.86 ± 1.13	19.96 ± 1.12	0.333
Gender, female, *n* (%)	681 (43.4)	94 (40.3)	485 (43.2)	102 (47.7)	0.289
Only child, *n* (%)	637 (40.6)	79 (33.9)	451(40.2)	107 (50.0)	0.002^**^
Rural area, *n* (%)	544 (34.7)	91 (39.1)	400 (35.7)	53 (24.8)	0.001^**^
Paternal education level (tertiary), *n* (%)	580 (37.0)	81 (34.8)	407 (36.3)	92 (43.0)	0.132
Maternal education level (tertiary), *n* (%)	483 (30.8)	66 (28.3)	344 (30.7)	73 (34.1)	0.410
Family income (≥5,000 yuan/month), *n* (%)	1,265 (80.6)	181 (77.7)	896 (79.9)	188 (87.9)	0.012^*^
**Lifestyle and health condition**
BMI (≥25 kg/m^2^), *n* (%)	106 (6.8)	18 (7.7)	72 (6.4)	16 (7.5)	0.695
Habitual napping (≥3 days/week), *n* (%)	1,477 (94.1)	220 (94.4)	1,060 (94.5)	197 (92.1)	0.378
Habitual snoring (≥3 days/week), *n* (%)	505 (32.2)	64 (27.5)	373 (33.2)	68 (31.8)	0.227
Boarding in school, *n* (%)	1,560 (99.4)	231 (99.1)	1,117 (99.6)	212 (99.1)	0.565
TV/Internet (≥4 h/day), *n* (%)	473 (30.1)	49 (21.0)	339 (30.2)	85 (39.7)	<0.001^***^
Mobile phone use before sleep (≥1 h/day), *n* (%)	303 (19.3)	33(14.2)	197(17.6)	73(34.1)	<0.001^***^
Smoking, *n* (%)	17 (1.1)	1 (0.4)	15 (1.3)	1 (0.5)	0.307
Drinking, *n* (%)	550 (35.1)	68 (29.2)	393 (35.0)	89 (41.6)	0.023^*^
Chronic medical conditions, *n* (%)	67 (4.3)	14 (6.0)	39 (3.5)	14 (6.5)	0.046^*^
High study pressure, *n* (%)	321 (20.5)	51 (21.9)	220 (19.6)	50 (23.4)	0.386
Low interest in learning, *n* (%)	1,090 (69.5)	127 (54.5)	786 (70.1)	177 (82.7)	<0.001^***^

*Chronotypes: E-types: reduced Morningness-Eveningness Questionnaire ≤11; I-types: 12 ≤reduced Morningness-Eveningness Questionnaire ≤17; M-types: reduced Morningness-Eveningness Questionnaire ≥18;*

* *p < 0.05*;

** *p < 0.01*;

*** *p <0.001*.

### Sleep Patterns, Sleep Disturbances, and Mental Distress Among Different Chronotypes

As shown in Table 2, significant differences were found in time in bed and sleep-wake habits among three groups both on weekdays and weekends (*p* <0.001). There were significant differences among the three on weekday bedtime and get-up time, weekend bedtime and get-up time, as well as sleep compensation during weekends (*p* <0.001). E-types had later bedtime, shorter weekdays time in bed, and more sleep compensation than M-types, and I-types showed a sleep pattern between these two extremes. In addition, E-types had significantly higher scores in ISI, BDI-13, and SAS when compared with M-types and I-types (all *p* <0.001). However, there was no difference in ESS total score among the three chronotypes (*p* >0.05).

**Table 2:** Comparisons of sleep patterns, sleep disturbances, and mental distress among different chronotypes.

**Characteristic**		**Total (*n* = 1,569)**	**M-types (*n* = 233)**	**I-types (*n* = 1,122)**	**E-types (*n* = 214)**	***P***	***Post*−*hoc*^a^**
**Sleep patterns** ^a^
Weekday	Bedtime (hh:mm)	00:06 ± 00:46	23:40 ± 00:43	00:05 ± 00:40	00:41 ± 00:53	<0.001^***^	M < I < E
	Get-up time (hh:mm)	07:28 ± 00:36	07:10 ± 00:48	07:28 ± 00:43	07:49 ± 00:36	<0.001^***^	M < I < E
	Time in bed (h)	7.36 ± 0.79	7.5 ± 0.88	7.37 ± 0.72	7.12 ± 0.88	<0.001^***^	M = I>E
Weekend	Bedtime (hh:mm)	00:26 ± 00:58	23:55 ± 00:56	00:23 ± 00:50	01:17 ± 01:06	<0.001^***^	M < I < E
	Get-up time (hh:mm)	8:49 ± 01:14	08:03 ± 01:23	08:45 ± 01:02	09:56 ± 01:15	<0.001^***^	M < I < E
	Time in bed (h)	8.37 ± 1.15	8.13 ± 1.45	8.37 ± 1.07	8.65 ± 1.12	<0.001^***^	M < I < E
Sleep compensation (h)		0.92(0.25, 1.58)	0.5 (0, 1)	0.9(0.3,1.5)	1.5 (0.82, 2)	<0.001^***^	M < I < E
**Sleep disturbances** ^b^
ISI total score		4 (2, 7)	3 (2, 6)	4 (2, 7)	5.5 (3, 9)	<0.001^***^	M < I < E
ESS total score		7 (5,10)	7 (4, 9)	7 (5, 9)	8 (4, 10)	0.116	
**Mental distress** ^b^
BDI-13 total score		3 (0,7)	2 (0, 5.5)	3 (0, 6)	5 (1, 10)	<0.001^***^	M = I < E
SAS total score		38.75 (35.00, 43.75)	40 (35.00, 43.75)	38.75 (35.00, 42.50)	41.25 (36.25, 46.25)	<0.001^***^	I < M < E

a *Statistical differences among three or more groups were determined by one-way ANOVA, followed by LSD post-hoc analysis*.

b *Statistical analysis was performed by Kruskal–Wallis one-way ANOVA and the Bonferroni post-hoc test for multiple comparisons*.

*M, M-types; I, I-types; E, E-types; ISI, Insomnia Severity Index; ESS, Epworth Sleepiness Scale; BDI-13, Beck Depression Inventory-13; SAS, Zung Self-Rated Anxiety Scale*.

*** *p < 0.001*.

### Comparisons of Sample Characteristics in Participants With Sleep Compensation, Sleep Disturbances, and Mental Distress

The relationships between the characteristics of participants and sleep compensation, sleep disturbances, and mental distress are presented in Tables 3, 4. In socio-demographic factors, age was related to sleep compensation and insomnia symptoms (*p* <0.05), and gender was related to sleep compensation, excessive daytime sleepiness (EDS), depressive and anxiety symptoms (*p* <0.001). In addition, rural areas and family incomes (≥5,000 yuan/month) were significantly associated with EDS (*p* <0.05), while parents' education level and family incomes (≥5,000 yuan/month) were significantly associated with depressive symptoms (*p* <0.05). With respect to lifestyle and health condition, time spent on TV or Internet (≥4 h/day), duration of mobile phone use before sleep (≥1 h/day), chronic medical conditions, high study pressure, low interest in learning were correlated with sleep compensation, sleep disturbances, and mental distress (*p* <0.05), except for duration of mobile phone use before sleep with EDS (*p* =0.238) and chronic medical conditions for sleep compensation (*p* =0.09). In addition, habitual napping (≥3 days/week) and smoking were correlated with depressive symptoms (*p* <0.05). What is more, chronotypes were correlated with sleep compensation, insomnia, depressive and anxiety symptoms (*p* <0.001), while there was no significant difference between chronotypes and EDS (*p* =0.107). When compared with M-types and I-types, E-types were associated with long weekend sleep compensation as well as more insomnia symptoms, EDS, and depressive symptoms (*p* <0.05), while there was no significant difference between M-types and I-types in these outcomes (*p* <0.05). Significant differences only existed between I-types and E-types in anxiety symptoms after Bonferroni correction. Those characteristics significantly related to sleep compensation, sleep disturbances, and mental distress were further controlled in multivariate analyses for chronotypes in these problems.

**Table 3:** Comparisons of sample characteristics in participants with and without sleep compensation and sleep disturbances.

	**Total** **(*n* = 1,569)**	**Short weekend sleep compensation (*n* = 1,292)**	**Long weekend sleep compensation (*n* = 277)**	***P***	**Without insomnia symptoms (*n* = 1,241)**	**With** **insomnia symptoms (*n* = 328)**	**P**	**Without excessive daytime sleepiness (*n* = 1,176)**	**With excessive daytime sleepiness (*n* = 393)**	***P***
**Socio-demographics**										
Age, year, mean ± SD	19.86 ± 1.16	19.80 ± 1.13	20.15 ± 1.21	0.019^*^	19.81 ± 1.09	20.03 ± 1.35	0.040^*^	19.87 ± 1.31	19.84 ± 1.23	0.659
Gender, female, *n* (%)	681 (43.4)	704 (54.5)	184 (66.4)	<0.001^***^	694 (55.9)	194 (59.1)	0.295	636 (54.1)	252 (64.1)	0.001^**^
Only child, *n* (%)	637 (40.6)	518 (40.1)	119(43.0)	0.784	735 (59.2)	197 (60.1)	0.784	691 (58.8)	241 (61.3)	0.37
Rural area, *n* (%)	544 (34.7)	448 (34.7)	96 (34.7)	0.717	427 (34.4)	117 (35.7)	0.911	388 (21.9)	156 (39.7)	0.049^*^
Paternal education level (tertiary), *n* (%)	580 (37.0)	482 (37.3)	98 (35.4)	0.546	461 (37.1)	119 (36.3)	0.772	441 (37.5)	139 (35.4)	0.449
Maternal education level (tertiary), *n* (%)	483 (30.8)	403 (31.2)	80 (28.9)	0.450	385 (31.0)	98 (29.9)	0.689	377 (32.1)	106 (27.0)	0.059
Family income (≥5,000 yuan/month), n (%)	1,265 (80.6)	1,046 (81.0)	219 (79.1)	0.468	1,006 (81.1)	259 (79.0)	0.392	963 (81.9)	302 (76.8)	0.029^*^
**Lifestyle and health condition**										
BMI (≥25 kg/m^2^), n (%)	106 (6.8)	88 (6.8)	18 (6.5)	0.815	82 (6.6)	24 (7.3)	0.649	81 (6.9)	25 (6.4)	0.719
Habitual napping (≥3 days/week), *n* (%)	1,477 (94.1)	1,222 (94.6)	255 (92.1)	0.105	1,167 (94.0)	310 (84.5)	0.745	1,104 (93.9)	373 (94.9)	0.45
Habitual snoring (≥3 days/week), *n* (%)	505 (32.2)	419 (32.4)	86 (31.0)	0.655	408 (32.9)	97 (29.6)	0.225	375 (31.9)	130 (33.1)	0.662
Boarding in school, *n* (%)	1,560 (99.4)	1,286 (99.5)	274 (98.9)	0.216	1,283 (99.8)	322 (98.2)	0.001^**^	1,173 (99.7)	387 (98.5)	0.004^**^
TV/Internet (≥4 h/day), *n* (%)	473 (30.1)	370 (28.6)	103 (37.2)	0.005^**^	342(27.6)	131(39.9)	<0.001^***^	592(50.3)	222(56.6)	0.035^*^
Mobile phone use before sleep (≥1 h/day), *n* (%)	303 (19.3)	218 (16.9)	85 (30.7)	<0.001^***^	208 (16.8)	95 (29.0)	<0.001^***^	219 (18.6)	84 (21.4)	0.238
Smoking, *n* (%)	17 (1.1)	12 (0.9)	5 (1.8)	0.202	13 (1.0)	4 (1.2)	0.776	14 (1.2)	3 (0.8)	0.585
Drinking, *n* (%)	550 (35.1)	440 (34.1)	110 (39.7)	0.073	438 (35.3)	112 (34.1)	0.698	400 (34)	150 (38.2)	0.135
Chronic medical conditions, *n* (%)	67 (4.3)	50 (3.5)	17 (12.6)	0.090	50 (3.5)	17 (12.6)	<0.001^***^	39 (3.1)	28 (8.5)	<0.001^***^
High study pressure, *n* (%)	321 (20.5)	254 (19.7)	67 (24.3)	<0.001^***^	207 (16.7)	114 (34.8)	<0.001^***^	217 (18.5)	104 (26.5)	0.001^**^
Low interest in learning, *n* (%)	1,090 (69.5)	877 (67.9)	213 (76.9)	0.003^**^	828 (66.7)	262 (79.9)	<0.001^***^	790 (67.2)	300 (76.3)	0.001^**^
**Chronotypes**				<0.001^***^			<0.001^***^			0.107
M-types	233 (14.9)	209 (16.2)^a^	24 (8.7)^a^		187 (15.1)^a^	46 (14.0)^a^		178 (15.1)	55 (14.0)	
I-types	1,122 (71.5)	942 (72.9)^a^	180 (65.0)^a^		914 (73.7)^a^	208 (63.5)^a^		850 (72.3)	272 (69.2)	
E-types	214 (13.6)	141 (10.9)^b^	73 (26.4)^b^		140 (11.3)^b^	74 (22.6)^b^		148 (12.6)	66 (16.8)	

*Short weekend sleep compensation: sleep compensation <2 h; Long weekend sleep compensation: sleep compensation ≥2 h; Insomnia symptoms: Insomnia Severity Index ≥8; Excessive daytime sleepiness: Epworth Sleepiness Scale ≥10*.

* *p < 0.05*,

** *p < 0.01*,

*** *p < 0.001*.

a, b *There were significant differences between different letters within each column after Bonferroni correction*.

**Table 4:** Comparisons of sample characteristics in participants with and without mental distress.

	**Total** **(*n* = 1,569)**	**Without depressive symptoms** **(*n* = 970)**	**With depressive symptoms** **(*n* = 599)**	***P***	**Without anxiety symptoms** **(*n* = 1,434)**	**With** **anxiety symptoms** **(*n* = 135)**	***P***
**Socio-demographics**							
Age, year, mean ± SD	19.86 ± 1.16	19.83 ± 1.18	19.93 ± 1.16	0.093	19.86 ± 1.15	19.91 ± 1.23	0.621
Gender, female, *n* (%)	681 (43.4)	520 (53.6)	368 (61.4)	0.002^**^	796 (55.5)	92 (68.1)	0.005^**^
Only child, *n* (%)	637 (40.6)	560 (57.7)	372 (61.1)	0.087	585 (40.8)	52 (38.5)	0.607
Rural area, *n* (%)	544 (34.7)	322 (33.2)	222 (37.1)	0.123	303 (21.1)	35 (25.9)	0.432
Paternal education level (tertiary), *n* (%)	580 (37.0)	380 (39.2)	200 (33.4)	0.021^*^	529 (36.9)	51 (37.8)	0.838
Maternal education level (tertiary), *n* (%)	483 (30.8)	319 (32.9)	164 (27.4)	0.022^*^	444 (31.0)	39 (28.9)	0.618
Family income (≥5,000 yuan/month), *n* (%)	1,265 (80.6)	802 (82.7)	463 (77.3)	0.009^**^	1,157 (80.7)	108 (80.0)	0.848
**Lifestyle and health condition**							
BMI (≥25 kg/m^2^), *n* (%)	106 (6.8)	62 (6.4)	44 (7.3)	0.465	97 (6.8)	9 (6.7)	0.966
Habitual napping (≥3 days/week), *n* (%)	1,477 (94.1)	924 (95.3)	553 (92.3)	0.016^*^	1,354 (94.4)	123 (91.1)	0.118
Habitual snoring (≥3 days/week), *n* (%)	505 (32.2)	306 (31.5)	199 (33.2)	0.49	470 (32.8)	35 (25.9)	0.103
Boarding in school, *n* (%)	1,560 (99.4)	965 (99.5)	595 (99.3)	0.698	1,427 (99.5)	133 (98.5)	0.144
TV/Internet (≥4 h/day), n (%)	473 (30.1)	225 (26.3)	218 (36.4)	<0.001^***^	420 (29.3)	53 (39.3)	0.016^*^
Mobile phone use before sleep (≥1 h/day), *n* (%)	303 (19.3)	149 (15.4)	154 (25.7)	<0.001^***^	255 (17.8)	48 (35.6)	<0.001^***^
Smoking, *n* (%)	17 (1.1)	6 (0.6)	11 (1.8)	0.024^*^	14 (1.0)	3 (2.2)	0.175
Drinking, *n* (%)	550 (35.1)	327 (33.7)	223 (37.2)	0.156	501 (34.9)	49 (36.3)	0.752
Chronic medical conditions, *n* (%)	67 (4.3)	22 (2.3)	45 (7.5)	<0.001^***^	50 (3.5)	17 (12.6)	<0.001^***^
High study pressure, *n* (%)	321 (20.5)	121 (12.5)	200 (33.4)	<0.001^***^	261 (18.2)	60 (44.4)	<0.001^***^
Low interest in learning, *n* (%)	1,090 (69.5)	601 (62.0)	489 (81.6)	<0.001^***^	985 (68.7)	105 (77.8)	0.028^*^
**Chronotypes**				<0.001^***^			<0.001^***^
M-types	233 (14.9)	157 (16.2)^a^	76 (12.7)^a^		212 (14.8)^a, b^	21 (15.6)^a, b^	
I-types	1,122 (71.5)	404 (74.0)^a^	404 (67.4)^a^		1,041 (72.6)^b^	81 (7.2)^b^	
E-types	214 (13.6)	95 (9.8)^b^	119 (19.9)^b^		181 (12.6)^a^	33 (15.4)^a^	

*Depressive symptoms: Beck Depression Inventory-13 ≥5; Anxiety symptoms: Zung Self-Rating Anxiety Scale ≥50*.

* *p < 0.05*,

** *p < 0.01*,

*** *p < 0.001*.

a, b *There were significant differences between different letters within each column after Bonferroni correction*.

### Multivariable Logistic Analyses

The binary logistic regression analysis results of the related factors of sleep disturbances and mental distress are shown in Table 5. After adjusting the socio-demographics, lifestyles, and health conditions, using the I-types as a reference, the E-types were found to indicate a higher risk of insomnia symptoms (adjusted OR 2.000, 95%CI 1.428–2.801), depressive symptoms (adjusted OR 2.068, 95%CI 1.496–2.858) and anxiety symptoms (adjusted OR 2.188, 95%CI 1.387–3.451). Compared with I-types, E-types were more likely to have long sleep compensation on weekends (adjusted OR 2.443, 95%CI 1.740–3.429), while M-types were the opposite (adjusted OR 0.623, 95%CI 0.392–0.990). Nonetheless, no significant associations were observed between chronotypes and EDS, except for E-types, which tended to have an association with EDS (*p* = 0.059).

**Table 5:** Associations of chronotypes with sleep disturbances and mental distress.

	**Coefficients B**	***P*-value**	**Adjusted OR^a^**	**95% confidence interval for OR**	
				**Lower**	**Upper**
**Long weekend sleep compensation**
M-types	−0.473	0.045^*^	0.623	0.392	0.990
E-types	0.893	<0.001^***^	2.443	1.740	3.429
**Insomnia symptoms**
M-types	0.163	0.392	1.177	0.810	1.710
E-types	0.693	<0.001^***^	2.000	1.428	2.801
**Excessive daytime sleepiness**
M-types	−0.003	0.984	0.997	0.708	1.403
E-types	0.319	0.059	1.375	0.987	1.915
**Depressive symptoms**
M-types	−0.064	0.702	0.938	0.675	1.303
E-types	0.727	<0.001^***^	2.068	1.496	2.858
**Anxiety symptoms**
M-types	0.178	0.504	1.195	0.708	2.016
E-types	0.783	0.001 ^**^	2.188	1.387	3.451

a *Logistic regression model controlled for age, sex, and other socio-demographics significantly correlated with sleep disturbances and mental distress, as well as lifestyles and health conditions with statistical significance (forward likelihood ratio method). I-types as the reference category. E-types: reduced Morningness-Eveningness Questionnaire ≤11; I-types: 12 ≤reduced Morningness-Eveningness Questionnaire ≤17; M-types: reduced Morningness-Eveningness Questionnaire ≥18; Long weekend sleep compensation: sleep compensation ≥2 hours; Insomnia symptoms: Insomnia Severity Index ≥8; Excessive daytime sleepiness: Epworth Sleepiness Scale ≥10. Depressive symptoms: Beck Depression Inventory-13 ≥5; Anxiety symptoms: Zung Self-Rating Anxiety Scale ≥50*.

* *p < 0.05*,

** *p < 0.01*,

*** *p < 0.001*.

## Discussion

This cross-sectional study attempted to investigate the distribution of chronotypes and the relationship between sleep disturbance and mental distress among college students. We identified that compared with I-types, E-types were positively associated with insomnia symptoms, sleep compensation, depressive symptoms, and anxiety symptoms, while M-types were negatively associated with sleep compensation.

In this study, we found that the most common chronotype of college students (71.5%) is I-type, which is consistent with previous studies (1, 34). In addition, the proportion of M-types (14.9%) was slightly higher than that of E-types (13.6%), which is consistent with a study in Hungary (35), but is different from an American study (36). Possible explanations for this discrepancy may include differences in the study sample size and population-related differences, such as the existence of socio-cultural diversity between the Chinese and Western populations.

We found that the E-type individuals were more likely to have sleep compensation on weekends, while those who were M-types were less likely to have sleep compensation on weekends. Several previous studies revealed that E-types generally go to bed and get up significantly later than M-types on both weekdays and weekends, which is consistent with our study (12, 37). Therefore, E-types are associated with a later bedtime and get-up time and a shorter time in bed during the weekdays when they are limited by the school schedule. However, on weekends, when there are less constrained by morning social demands, E-types may extend their sleep until a more favorable biological wake-up time to make up the deficit accumulated on weekdays (38). The need to get up at an earlier biological time to accommodate study and social demands on weekdays and frequently shift their sleep patterns between weekdays and weekends, producing a distinct phenomenon termed “social jetlag” (39, 40), might explain why E-types had more sleep compensation than other types.

This study also identified that E-types were more likely to have insomnia symptoms. Several previous studies revealed that E-type insomnia patients showed greater sleep-wake variability, more sleep-related dysfunctional cognitions, and less sleep hygiene knowledge than those with other chronotypes (41). It has also been hypothesized that E-types, with maladaptive sleep-related cognitions and irregular sleep schedules, might be a risk factor for perpetuating insomnia symptoms (42).

Our findings indicate a tendency toward a significant relationship between E-types and EDS, which is consistent with previous research that found a higher complaint of EDS among college students with E-types (43). Similarly, a previous study confirmed a significant association between M-types and lesser EDS (38). However, a previous study reported the absence of a correlation between chronotype and EDS among college students (44). The possible explanation for this observed discrepancy might be the different study populations, socio-demographic characteristics, and socio-cultural characteristics. The existing findings between chronotypes and EDS in college students remain inconclusive; further investigation is required.

With respect to mental distress, the finding of this study indicates that E-types were positively associated with depressive symptoms and anxiety symptoms of college students after controlling for other predictors, which is consistent with many previous studies (45–47). Similar findings showed that the E-type was correlated with depressive symptoms in Chinese college students and Dutch (48, 49). Many factors might contribute to the increased mental distress risks among E-types in a complex way. Previous research showed that the main mechanism underlying chronotype and mood problems involved variations in biological clock genes (CLOCK, PER1, and PER2) (50). Moreover, previous studies suggested that chronotypes and sleep disruption may play an important role in susceptibility to mental distress and the precipitation of disorder symptoms (51, 52). Furthermore, people with E-types may increase the risk of mental health problems, including depression and anxiety due to disturbance of the sleep and wake cycle with melatonin and serotonin deficiency (53, 54).

The findings of the present study have important clinical and public health implications. Intervention and prevention strategies should be directed to target both the E-type and mental health in the context of psychopathology. Several positive and effective measures should be taken to avoid delay of the circadian rhythm of E-types, such as reducing the use of electronic devices with luminous screens (i.e., game consoles, tablets, computers, and mobile phones) before bedtime. Furthermore, they are supposed to get enough early light exposure by going outside early in the morning, which may help stabilize and advance their circadian rhythms. Therefore, it is important for school and health care providers to provide sleep hygiene education and necessary psychological intervention in time so as to prevent further exacerbations when detecting students with sleep disturbances and mental distress. Further prospective studies are warranted to examine the efficacy of interventions and prevention programs for circadian factors in improving sleep and mental health problems.

The advantages of the present study lie in its large sample size, which can make our findings convincing. Furthermore, to our best knowledge, there are few studies investigating the chronotypes and their relationships with sleep disturbances and mental distress among Chinese college students. Our study also has some limitations. Firstly, this cross-sectional survey cannot determine the causal relationship between chronotypes and their correlates. Secondly, data were collected through self-reported questionnaires rather than objective measures; this might lead to recall bias and social desirability bias. Physiological measurements of circadian rhythms (i.e., actigraphy) are more accurate methods to assess the chronotype, but these methods are too challenging and expensive for such a large sample as in this study, while the use of self-report questionnaires by online surveys can collect a large amount of data efficiently within a comparatively short time. However, research showed a strong correlation between subjective and objective measures of chronotypes and sleep durations. In addition, it was unable to calculate a response rate because the accurate number of students who received the message of the link was unknown. Finally, all of the participants were recruited from a single college, which may limit the finding's generalizability.

## Conclusions

Our study showed a significant correlation between E-types and long weekend sleep compensation, symptoms of insomnia, depression, and anxiety among Chinese college students. Our findings emphasize the importance of early recognition and intervention of E-types and their accompanied sleep problems and mental distress.

## Data Availability Statement

The raw data supporting the conclusions of this article will be made available by the authors, without undue reservation.

## Ethics Statement

The studies involving human participants were reviewed and approved by the Ethics Committee of Nanfang Hospital, Southern Medical University (QTEC-2019-101). Written informed consent to participate in this study was provided by the participants' legal guardian/next of kin.

## Author Contributions

JW and BZ: conceptualization. JW, SL, and BZ: methodology. JW: writing—original draft. JW, SL, and JG: formal analysis. SL, RX, JY, XL, YX, YZ, YC, YG, LC, and BZ: investigation, resources, and data curation. SL, JG, and BZ: writing—review and editing. SL and BZ: funding acquisition. BZ: supervision and project administration. All authors have approved the final manuscript.

## Funding

This work was supported by the National Key R&D Program of China (Grant No. 2021YFC2501500), the National Natural Science Foundation of China (BZ: Grant No. 82071488 and SL: Grant No. 81901348), the Chinese Sleep Research Society Hansoh Project, China (Grant No. 2019HSC03), the President Foundation of Nanfang Hospital, Southern Medical University (BZ: Grant No. 2019Z014), and Education Research Projects of Nanfang Hospital (Grant No. 21NJ-ZDPY01).

## Conflict of Interest

The authors declare that the research was conducted in the absence of any commercial or financial relationships that could be construed as a potential conflict of interest.

## Publisher's Note

All claims expressed in this article are solely those of the authors and do not necessarily represent those of their affiliated organizations, or those of the publisher, the editors and the reviewers. Any product that may be evaluated in this article, or claim that may be made by its manufacturer, is not guaranteed or endorsed by the publisher.
